# Comprehensive analysis of metastatic gastric cancer tumour cells using single-cell RNA-seq

**DOI:** 10.1038/s41598-020-80881-2

**Published:** 2021-01-13

**Authors:** Bin Wang, Yingyi Zhang, Tao Qing, Kaichen Xing, Jie Li, Timing Zhen, Sibo Zhu, Xianbao Zhan

**Affiliations:** 1 Department of Oncology, Changhai Hospital, Second Military Medical University, No. 168 Changhai Road, Shanghai, 200433 China; 2 School of Life Sciences, Fudan University, No. 2005 Songhu Road, Shanghai, 200438 China; 3 Shanghai Cinoasia Institute, Shanghai, 200438 China

**Keywords:** Cell biology, Cancer, Gastrointestinal cancer

## Abstract

Gastric cancer (GC) is a leading cause of cancer-induced mortality, with poor prognosis with metastasis. The mechanism of gastric carcinoma lymph node metastasis remains unknown due to traditional bulk-leveled approaches masking the roles of subpopulations. To answer questions concerning metastasis from the gastric carcinoma intratumoural perspective, we performed single-cell level analysis on three gastric cancer patients with primary cancer and paired metastatic lymph node cancer tissues using single-cell RNA-seq (scRNA-seq). The results showed distinct carcinoma profiles from each patient, and diverse microenvironmental subsets were shared across different patients. Clustering data showed significant intratumoural heterogeneity. The results also revealed a subgroup of cells bridging the metastatic group and primary group, implying the transition state of cancer during the metastatic process. In the present study, we obtained a more comprehensive picture of gastric cancer lymph node metastasis, and we discovered some GC lymph node metastasis marker genes (ERBB2, CLDN11 and CDK12), as well as potential gastric cancer evolution-driving genes (FOS and JUN), which provide a basis for the treatment of GC.

## Introduction

Globally, gastric cancer (GC) is the fifth most common and one of the leading cancers that cause mortality. In 2012, there were 952,000 cases diagnosed, resulting in an estimated 723,000 annual deaths^1^. Gastric cancer represents a poor prognosis, and the total 5-year survival is less than 30%, despite treatment with surgery^2^. Lymphatic metastasis was expected to be associated with poor outcomes, and in poorer stages, more distant lymph nodes can be revealed through histopathology finding^3,4^. More accurate diagnosis and precision therapy are the priorities of current clinical and fundamental research in gastric cancer. Based on distinct molecular subtypes in The Cancer Genome Atlas (TCGA) Network, gastric cancer is now regarded to have specific genomic abnormalities and targeted therapies^5^.

Biologists and clinicians are faced with many challenges, including gastric neoplasm metastasis. Genomic analysis revealed a series of significant actionable mutation loads or pathways in gastric cancer, such as PI3K/AKT/mTOR, CLD18, and HER2/EGFR, which are likely to induce primary gastric cancer to develop into metastases^6,7^. Recently, transcriptomic data have revealed that the RhoA pathway is involved in the invasion and migration of the ‘diffuse’ growth phenotype in gastric cancer^8^. Metastatic cascades have been depicted by several steps, including the dissemination of circulating cells, adhesion to blood vessel endothelial cells and proliferation^9^. However, the mechanism of gastric carcinoma lymph node metastasis remains unknown, partly because data from metastasis studies were generated with the bulk approach, which were likely to mask the roles of subpopulations. Therefore, heterogeneity caused by diverse tumour cell subsets and complex microenvironments has been a great challenge in diagnosis and treatment.

Single-cell RNA-seq (scRNA-seq) deciphers tissues into individual cells to distinguish neoplastic from nontumourous cells and to profile expression patterns to infer subclones^10^. ScRNA-seq can be used to analyse metastatic cancer cells whose bulk-level expression profiles are affected by metastasized local tissue^11–13^. In the study of GC scRNA-seq, Li et al. found that some specific marker genes, including SLC11A2, KLK7 and SULT2B1, were related to the development of early GC cells^14^. Single-cell gene expression studies revealed widespread changes in cell numbers, transcriptional status, and intercellular interactions in the GC tumour microenvironment^15^. ScRNA-seq can be employed in analysing different degrees of GC, which is a potential good predictor of GC prognosis^16^. These studies have paid attention to the tumour heterogeneity of GC but lack research on GC metastasis. To answer questions from the GC intratumoural perspective in metastasis, we performed single-cell level analysis on three GC patients with primary cancer and paired metastatic lymph node cancer tissue using the scRNA-seq approach.

## Materials and methods

### Experimental design

**Figure 1:**
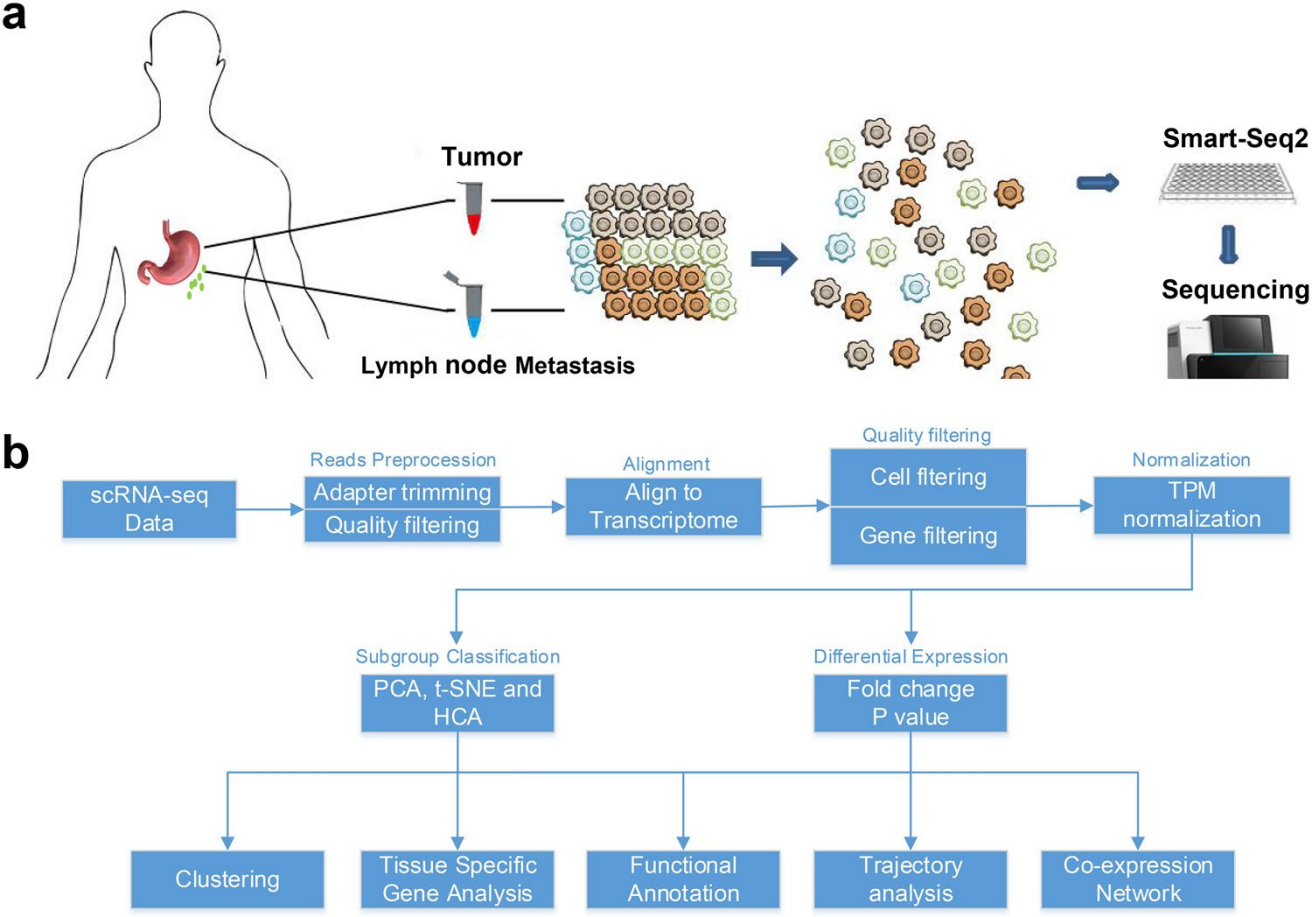
(**a**) Overview of the study design and sampling protocol. (**b**) Analysis pipeline in the current single-cell RNA-seq study.

The experiment was performed by comparative sequencing analysis using scRNA-seq from the primary tumour tissue (TT) and paired lymph node (LN) metastasis tumour tissue in three gastric cancer patients. The clinical characteristics of each patient used in this study are shown in Table 1. Tumour tissues were obtained from Changhai Hospital affiliated to Second Military Medical University during operations. The study was approved by the Ethical Committee of Changhai Hospital (CHEC2016-157). Informed consent was written by each of the patients and their guardians, and all procedures were conducted per the Helsinki Ethical Principles for Medical Research. All libraries were prepared with the Smart-seq2 scRNA-seq protocol and sequenced on a HiSeq2500 instrument with 50 bp single-end sequencing mode (Fig. 1).

**Table 1:** Clinical characteristics of each patient used in the scRNA-seq study and the cell number of each sample after quality control.

Clinical characteristics	Patient		
	PT1	PT2	PT3
Numbers of analysis cells (TT/LN)	19/4	27/13	19/12
Age	55	33	67
Sex	Male	Male	Male
Histopathological diagnosis	Moderately differentiated adenocarcinoma	Moderatly low differentiation adenocarcinoma	Moderately low differentiation adenocarcinoma
Pathological stage	IIIA	IIA	IIIB
T	1b	2	3
N	2	0	1
M	0	0	0
Site of origin	Gastric angle	Antrum	Cardia/GNor
Infiltration degree	Submucosal	Muscularis	Serosa
Tumour size (cm)	2.5 × 4 × 0.4	2.6 × 2.1 × 0.5	5.5 × 5.5 × 0.6
CEA (ng/mL)	2.20	2.68	2.88
CA724 (U/mL)	6.53	1.64	1.65
CA199 (U/mL)	< 2.20	14.93	< 2
HER2 IHC	3 +	Negative	1 +
HER2 FISH	Positive	Negative	Positive
HER2/CSP17	> 2	1.07	2.51
Ki67	80%	80%	80%
P53	Negative	10%	Negative

*PT* patient, *TT* tumour tissue, *LN* lymph node, *CEA* carcinoembryonic antigen, *CA* carbohydrate antigen, *CSP17* centromere-specific probe 17, *HER2* human epidermalgrowth factor receptor-2, *IHC* immunohistochemistry, *FISH* fluorescence in situ hybridization.

### Solid tumour decomposition and single cell isolation simulation

Biopsy or metastatic tumour were dissected and transferred to a 2 ml tube (Axygen, China), each containing 1 ml prewarmed M199 media (Thermo Fisher Scientific, USA), 2 mg/ml collagenase P (Roche, USA) and 10 U/µl DNase I (Roche, USA) as described by Tirosh et al.^17^. Tissues were digested for 60 min at 37 °C and then pipetted up and down every ten times every 10 min. The tissue suspensions were then filtered with a 70 µm nylon mesh (Thermo Fisher Scientific, USA) and centrifuged at 450*g* for 5 min. Pellets were resuspended for live cell staining using CFSE incubation for 5 min.

### Single-cell whole-transcriptome library preparation and sequencing

Single cells from each tissue were manually picked under fluorescence microscopy (X71, Olympus, Japan) using a mouth pipette. Each of the harvested single cells was transferred into 2 µL of cell lysis buffer (CLB) in 0.2 mL PCR tubes. Libraries of isolated single cells were then prepared as per the Smart-seq2 protocol^18^ with modifications on reverse transcription and amplification cycles.

Oligo-dT primed RT (reverse transcription) was performed by Smartscribe (Takara, Japan) reverse transcriptase and locked TSO oligonucleotide (Exiqon, Danmark) upon single cells. Full-length cDNA amplification was conducted by PCR amplification for 22 cycles with Hifi HotStart ReadyMix reagent (KAPA Biosystems, USA) and purified by 0.6 × AMPure beads (BD, USA). Barcoded libraries were fragmented and segmented with a Library Prep kit (Nextera XT, Illumina, USA). Pooled libraries with unique N5–N7 barcodes were sequenced with a HiSeq 2500 sequencer (Illumina, USA) and a 50 SE read flow cell.

### ScRNA-seq data analysis

Sequencing adapters and low-quality reads were first trimmed and removed using Trimmomatic^19^. Reads with a Phred score below 20 and trimmed sequence lengths less than 18 bp were discarded. The remaining high-quality reads were mapped to the human genome using the HiSat2 tool^20^ by using the human genome UCSC hg19 as a reference (ftp://genome-ftp.cse.ucsc.edu/goldenPath/hg19/chromosomes/) with a total of 22,335 genes. FeatureCounts software^21^ was used for the expression calculation of each gene, and raw count values of genes in each sample were obtained. A gene that was considered to be expressed in a sample had one more count in the sample. Read counts were normalized to TPM (transcript per million) values and then log2 transformed by using the “newSCESet” function of “scater” (https://github.com/davismcc/scater) package by R (https://www.r-project.org/).

Analyses, including principal component analysis (PCA), Pearson correlation, Student’s t-test and hierarchical clustering analysis (HCA), were performed using functions in R as follows: ‘prcomp’, ‘cor’, ‘t.test’ and ‘cluster’ in the ‘stats’ package and Heatmap in the ‘ComplexHeatmap’ package. The “ggplot” was used for the visualization of PCA.

Differentially expressed genes (DEGs) were calculated with fold-change and p-value between "treatment" and control groups. We set the fold change by a twofold cut-off, and FDR-adjusted p < 0.05 was regarded as the criterion for DEGs. This was carried out by using the “stat” package. Gene Ontology (GO) analysis results were obtained based on the Metascape (http://metascape.org)^22^.

Single-cell trajectory analysis. We used TSCAN, diffusion map, and monocle2 to perform pseudotime trajectory analysis for the evolution of gastric cancer cells. Cells were chosen based on Seurat cluster identification results.

### Immunofluorescence

GC tumour tissues were embedded in paraffin and sliced by Servicebio (Shanghai, China). The antibodies were purchased from Abcam (Abcam, Cambridge, UK), including Anti-ERBB2 and Anti-Oligodendrocyte Specific Protein (CLDN11).

## Results

### Sequencing data processing and QC

After filtration with a per-gene average read > 1 across all samples, 94 out of 171 samples passed quality control (Table 1). A total of 7601 genes passed filtration and were adopted in further analysis. Each cell was sequenced with 20,000 ~ 200,000 uniquely mapped reads, which is sufficient to detect distinct subpopulation expression profiles^17,23–25^. Correlations between individual tumour cells from different samples showed a broad range of Pearson coefficients (r = − 0.1 ~ 0.98), implying prominent transcriptomic heterogeneity. However, despite the heterogeneity across the cells, most of the samples were clustered according to their tissue of origin (Figure S1).

### Clustering of the primary tumour and metastatic tumour

**Figure 2:**
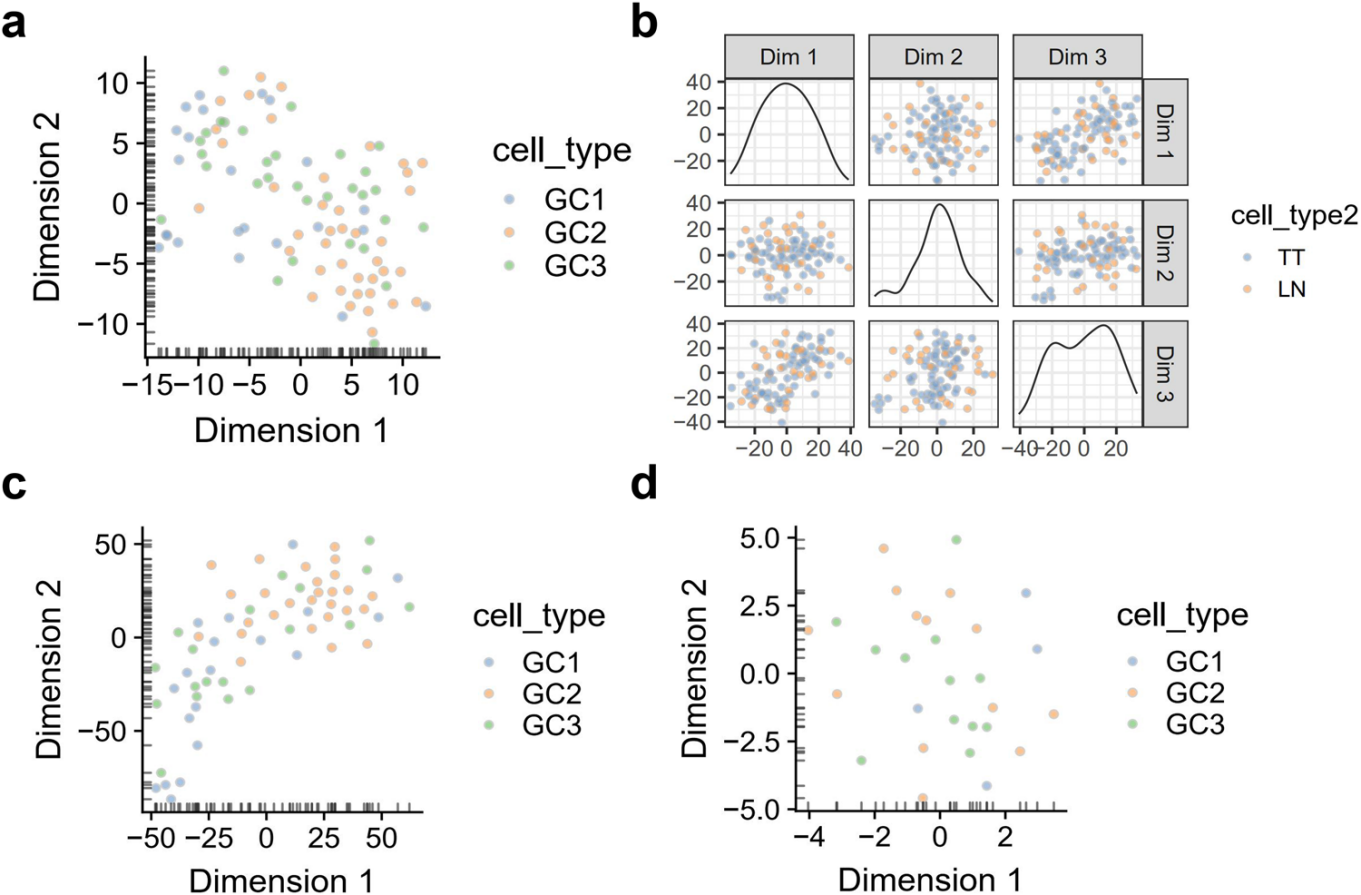
(**a**) T-SNE was plotted to present the distribution of the single cells from three patients in all primary tumour tissues. (**b**) Unsupervised T-SNE showing the separation of carcinoma cell groups. (**c**) In terms of gastric cancer tumour tissues, removal of noncarcinoma cells reveals intrinsic patient-specific tumour cell heterogeneity. (**d**) More randomly dispersed dots are shown in the metastatic tumour cells.

T-SNE was plotted to present the distribution of the single cells from the primary tumour and metastatic tumour in lower dimensions. Primary and metastatic tumour subgroups were partly merged (Fig. 2). Unsupervised T-SNE showed the separation of the primary tumour and metastatic tumour cell groups. In terms of tumour tissues, removal of nontumourous cells resulted in distinct patient-specific cancer heterogeneity. ScRNA-seq revealed specific carcinoma subpopulations and their characteristics from each patient. However, diverse microenvironmental populations were shared by the different patients, and nonmalignant cells did not show clustering of any specific subgroups.

### Intratumoural heterogeneity analysis

**Figure 3:**
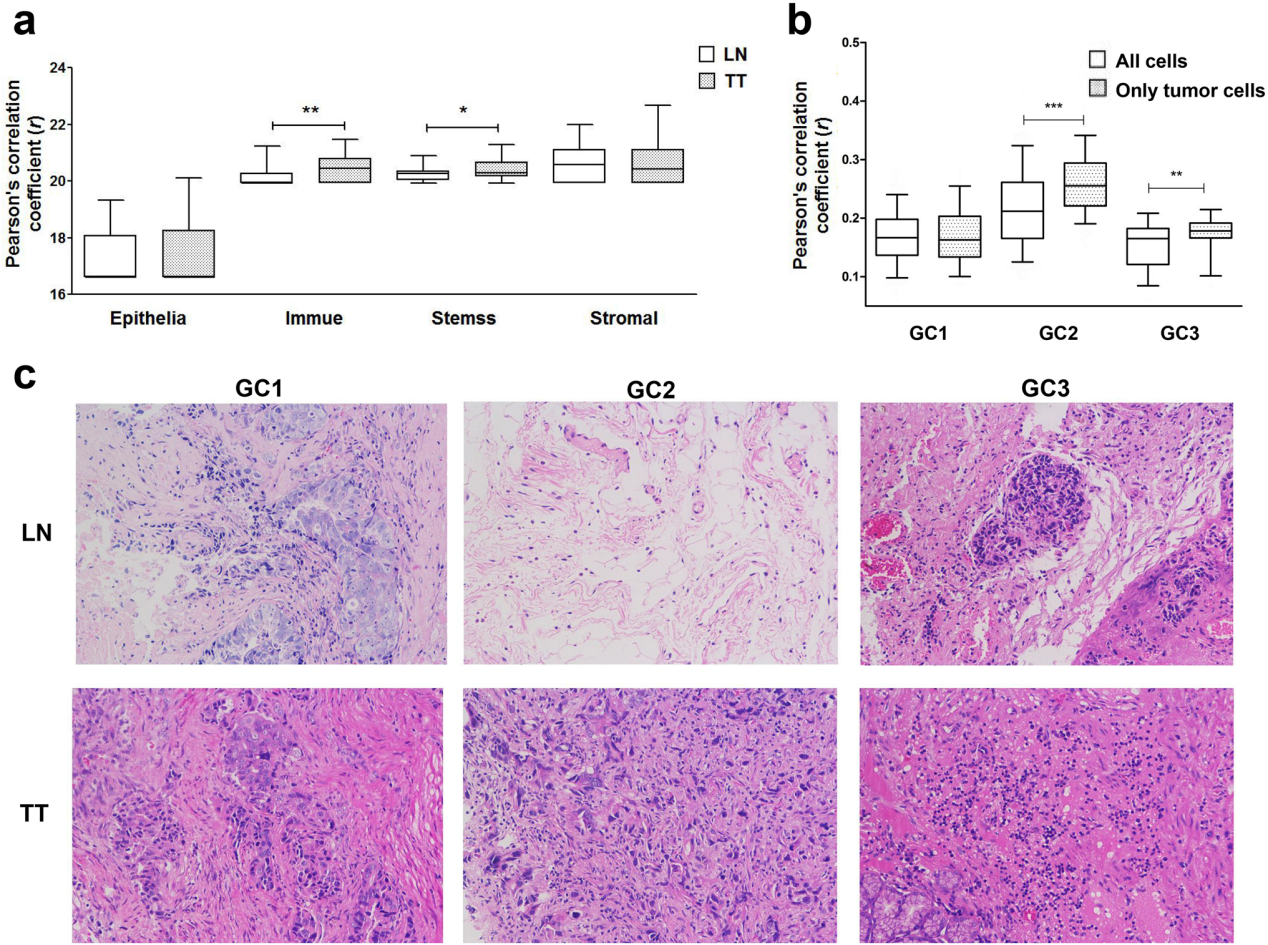
Intratumoural heterogeneity analysis. Correlation analysis of single cells revealed heterogeneity within tumours across three patients. Robust bulk stemness, immune, stromal, and tumour scoring assessment between tumour and paratumour single cells.

**Figure 4:**
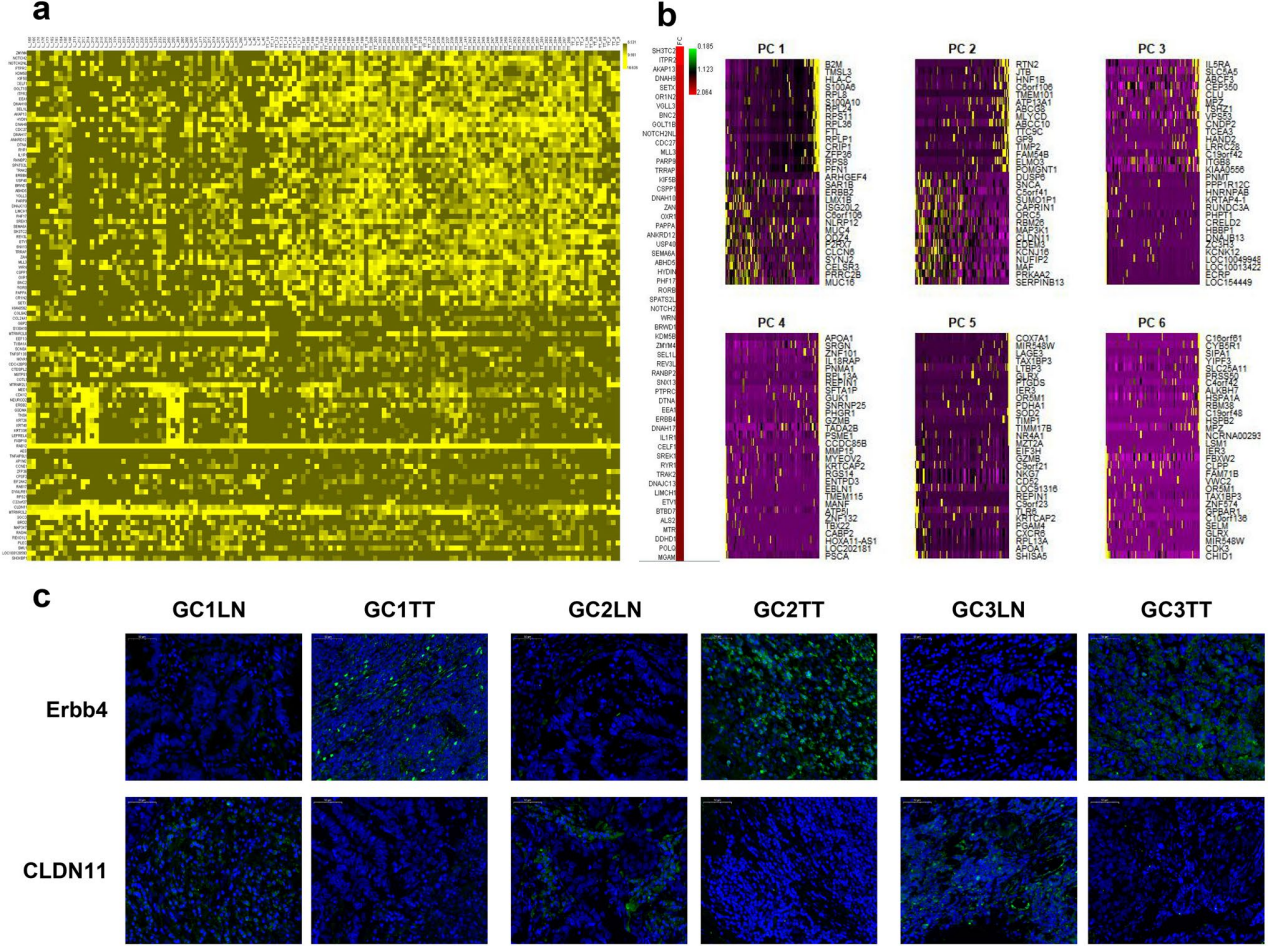
Tissue-specific markers. (**a**) Population-wide comparison between TT and LN single cells. Tissue-specific markers were calculated, and a heatmap was plotted using the top 100 highly expressed features based on previously defined clusters. NOTCH2, NOTCH2NL, KIF5B, and ERBB4 are highly expressed in primary cancer, while CDK12, ERBB2, and CLDN11 are overexpressed in metastatic cancer. Functional annotation revealed microtubule movement, and notch-based signalling was activated in the primary cells, indicating its metastatic propensity. (**b**) Decomposition of six main principal components (PCs) in the datasets. (**c**) IF of ERBB4 and CLDN11 in TT and LN.

In terms of the intratumoural heterogeneity, the correlation analysis of single cells revealed heterogeneity within tumours across three patients (Fig. 3). Using bulk stemness, immune, stromal, and tumour scoring assessments, we found significant tumour and stromal scoring differences between primary tumour and metastatic tumour single cells, indicating compositional and functional changes in tumours. Population-wide comparison between TT and LN single cells revealed that NOTCH2, NOTCH2NL, KIF5B, and ERBB4 are highly expressed in primary cancer, while CDK12, ERBB2, and CLDN11 are overexpressed in metastatic cancer. The decomposition of four main principal components (PCs) in the datasets is shown in Fig. 4.

### ScRNA-seq analysis and trajectory analysis of cell clusters

**Figure 5:**
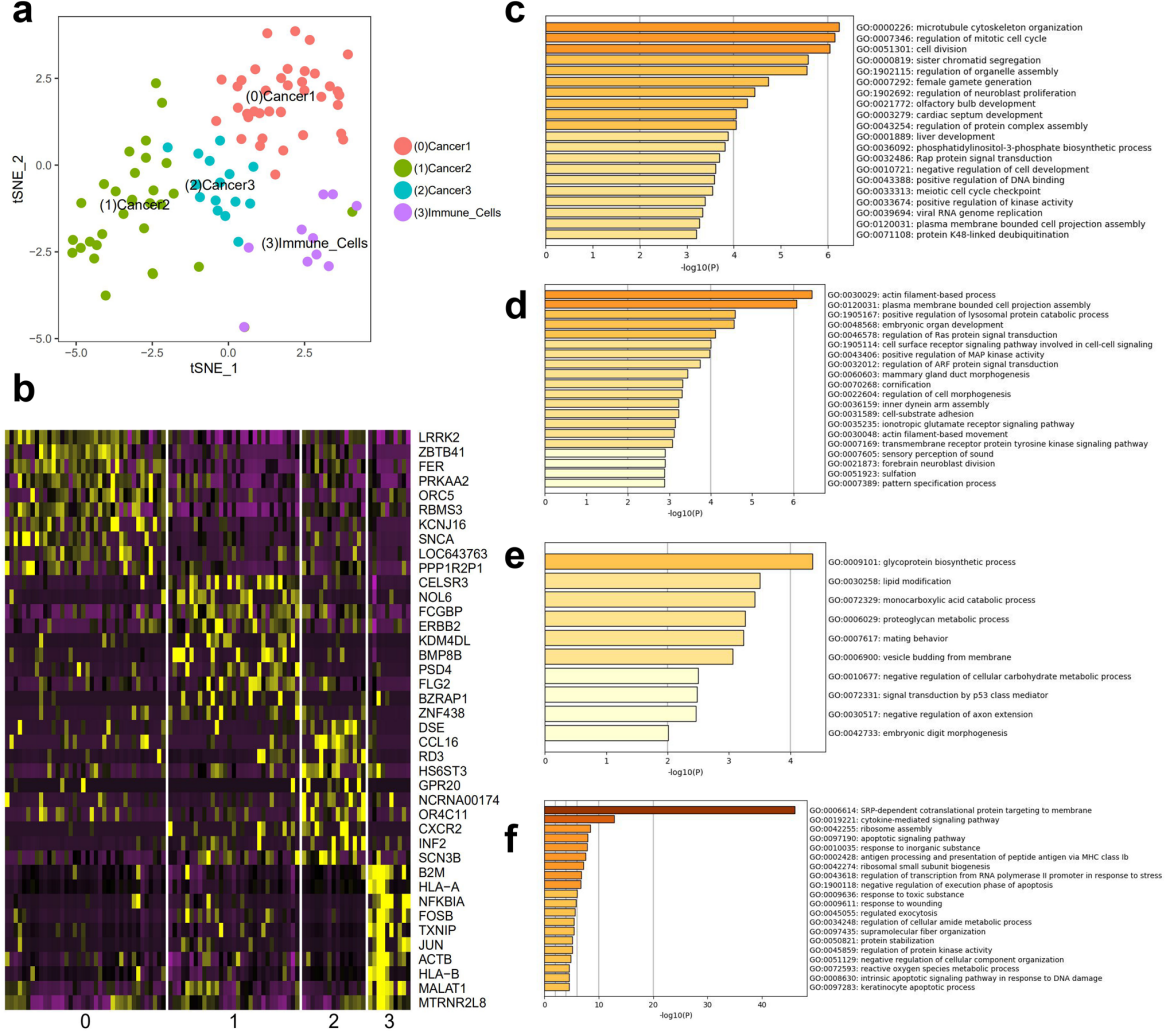
Seurat marker analysis revealed four main clusters in the overall single cells. Twelve significant principal components were extracted to identify four main clusters in the tumour tissues. (**a**) Four main clusters in the tumour tissues using TSNE. (**b**) Heatmap indicating markers highly expressed in each cluster. (**c**–**f**) Functional annotations of each cluster are shown based on Seurat-calculated markers.

Seurat marker analysis revealed four main clusters in the overall single cells. Twelve significant principal components were extracted to identify four main clusters in the tumour tissues. The heatmap indicates markers highly expressed in each cluster. Functional annotations of each cluster are shown based on Seurat-calculated markers (Fig. 5).

**Figure 6:**
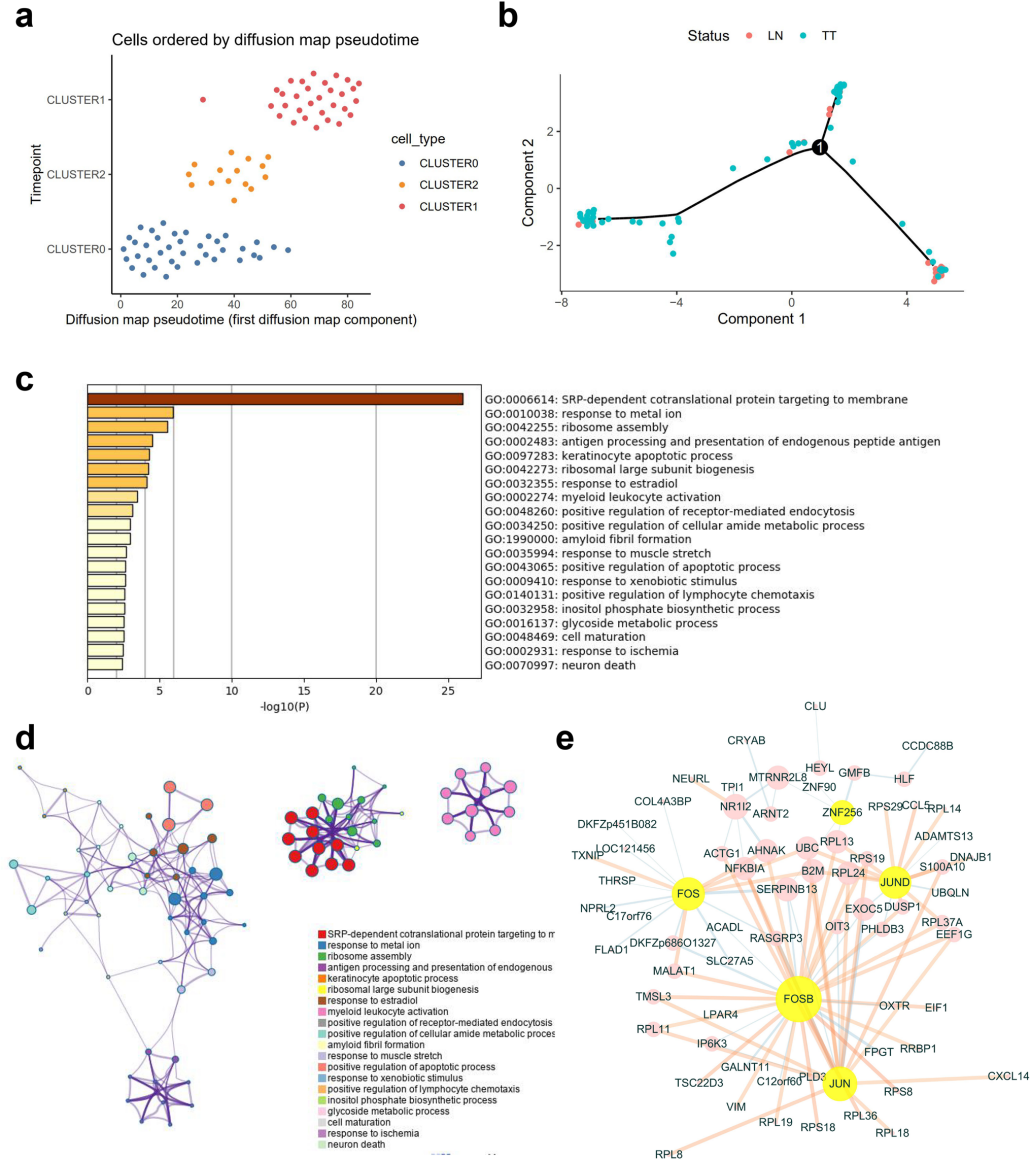
Gastric-derived cell evolutionary trajectory. (**a**) The pseudotime trajectory of GC clusters revealed a distinct pattern of postulated evolution state from Cluster 0 > 2 > 1. (**b**) The evolutionary trajectory of TT and LN cells. (**c**,**d**) Evolution trajectory-based functional annotation (Top1000 gene). (**e**) Regulatory co-network of kernel genes in evolution regulation and a transcription factor-driven regulatory network.

The pseudotime trajectory of GC clusters revealed a distinct pattern of postulated evolution state from Cluster0 > 2 > 1. The major genes (TOP1000) driving evolution were mainly involved in SRP-dependent cotranslational protein targeting to the membrane, response to the metal ion, and ribosome assembly. The kernel genes in evolution regulation include SERPINB13, NFKBIA, B2M, and RPL24. Transcription factors including FOS, FOSB, JUN, JUNB, and ZNF256 drive the regulatory networks (Fig. 6).

Stem cell markers were applied to validate the multiple GC origin hypotheses. Based on canonical markers, we identified hepatocytes and macrophage cells using TSNE. Using TSCAN, we postulated a gastric-derived cell evolutionary trajectory. SLICER, TSCAN, and diffusion map pseudotime tools show the evolutionary trajectory of four tumour cell clusters (Figure S2).

## Discussion

Tumour heterogeneity in gastric tumorigenesis and progression has recently attracted researchers’ attention. Based on some significant findings and theories of heterogeneity, target and immune therapies are in progress. However, there remains largely unknown heterogeneity in gastric cancer. By using single-cell isolation aided scRNA-seq, we could clearly identify the signatures of the primary tumour and metastatic tumour and reveal the role of heterogeneity, which causes metastasis in gastric cancer. Gastric carcinoma is common in lymph node metastasis, but the mechanism remains unknown. In our study, we revealed a subgroup of cells bridging the metastatic group and primary group, implying the transition state of cancer during the metastatic process by analysis of three patients’ transcriptomic data of single cells from primary gastric tumours and lymph node metastasis tumours.

Cancer heterogeneity has been shown to be a great challenge in cancer diagnosis and treatment^26^. Recently, scRNA-seq has been able to analyse abnormal cell-to-cell interactions, chemotherapy resistance, and immunosuppressive microenvironments from primary tissues or CTCs. For example, Chun et al*.* separated tumour cells and immune cells from primary breast cancer cells^27^. Patel et al*.* revealed unanticipated heterogeneity in primary glioblastoma, showing diverse regulatory signalling and therapy programmes^28^. Kim et al*.*’s scRNA-seq results combined both intratumoural SNV KRAS^G12D^ and expression heterogeneity of lung adenocarcinoma cells, deciphering subpopulations in anticancer drug responses^29^. In terms of the intratumoural cancer cell component. Our clustering data showed significant intratumoural heterogeneity.

Tissue-specific markers were calculated, and a heatmap was plotted using the top 50 highly expressed features based on six redefined clusters using cell markers. Tissue-specific markers were calculated, and a heatmap was plotted using the top 100 highly expressed features based on previously defined clusters. NOTCH2, NOTCH2NL, KIF5B, and ERBB4 are highly expressed in primary cancer, while ERBB2, CLDN11 and CDK12 are overexpressed in metastatic cancer. Previous studies suggested that the expression of Notch signalling pathway-associated proteins, such as Notch2, was significantly elevated in gastric cancer tissues compared to normal tissues^30^. In addition, evidence showed that higher KIF5B and ERBB4 promoted cancer cell proliferation^31,32^. Evidence suggests that CDK12, ERBB2, and CLDN11 play an associated metastatic role in cancer^33–35^. Several studies have shown that CLDN11 is related to tumour migration and metastasis^35^. Although the aetiologies of gastric cancer are partly clear and validated by experiments, the proposed “seed”-and- “soil” hypothesis has yet to be well explained^36^. Our findings implied that lymph node metastasis-prone subclones are more likely to share CLDN11, which is a member of the tight junction protein family that functions as a component of cell adhesion^37^. This explained cancer cell colonization in the lymph node after migrating from primary tissues. Future studies need to evaluate both transcriptomic and genetic alterations and even geographical information across different regions from primary metastatic tumours only to identify survival and evolution pressure in the cancer-related microenvironment, which promotes the identification of potential key drivers of gastric cancer.

TFs are proteins with special structures and functions that regulate gene expression. We noticed that several TF-regulating genes, including FOS and JUN, appeared to be particularly important during tumour evolution. JUN and FOS were determined to be critical genes related to GC^38^. ScRNA-seq reveals an expression pattern with high FOS and JUN at leukaemia evolution, which resolves following therapy but reoccurs following relapse and death^39^.

As this is a preliminary study, the limitation of our analysis is the small number of patient cases enrolled in this study. Nonetheless, we obtained a more comprehensive picture of gastric cancer lymph node metastasis at single-celled resolution, giving a new perspective on the biomarkers (ERBB2, CLDN11 and CDK12) involved in metastasis, pathways involved and driver genes (FOS and JUN) during the metastasis process, providing a basis for the treatment of GC.

## Supplementary Information

Supplementary Figures.

## Data Availability

Raw data of scRNA-seq were deposited in GEO (GSE158631).

## References

[1] McGuire, S. World Cancer Report (2014). Geneva, Switzerland: World Health Organization, International Agency for Research on Cancer, WHO Press, 2015. Adv. Nutr..

[2] Aurello P (2007). Classification of lymph node metastases from gastric cancer: Comparison between N-site and N-number systems. Our experience and review of the literature. Am. Surgeon.

[3] Grimes JA (2017). Agreement between cytology and histopathology for regional lymph node metastasis in dogs with melanocytic neoplasms. Vet. Pathol..

[4] Ehteshami Bejnordi B (2017). Diagnostic assessment of deep learning algorithms for detection of lymph node metastases in women with breast cancer. JAMA.

[5] Cislo M (2018). Distinct molecular subtypes of gastric cancer: From Lauren to molecular pathology. Oncotarget.

[6] Brastianos PK (2015). Genomic characterization of brain metastases reveals branched evolution and potential therapeutic targets. Cancer Discov..

[7] Nguyen DX (2009). WNT/TCF signaling through LEF1 and HOXB9 mediates lung adenocarcinoma metastasis. Cell.

[8] Yoon C (2016). Chemotherapy resistance in diffuse-type gastric adenocarcinoma is mediated by RhoA activation in cancer stem-like cells. Clin. Cancer Res..

[9] Valastyan S, Weinberg RA (2011). Tumor metastasis: Molecular insights and evolving paradigms. Cell.

[10] Muller S, Diaz A (2017). Single-cell mRNA sequencing in cancer research: Integrating the genomic fingerprint. Front. Genet..

[11] Hanahan D, Weinberg RA (2011). Hallmarks of cancer: The next generation. Cell.

[12] Neal JT (2018). Organoid modeling of the tumor immune microenvironment. Cell.

[13] Bancells C (2019). Revisiting the initial steps of sexual development in the malaria parasite *Plasmodium falciparum*. Nat. Microbiol..

[14] Zhang P (2019). Dissecting the single-cell transcriptome network underlying gastric premalignant lesions and early gastric cancer. Cell Rep..

[15] Sathe A (2020). Single-cell genomic characterization reveals the cellular reprogramming of the gastric tumor microenvironment. Clin. Cancer Res..

[16] Zhang M (2020). Dissecting transcriptional heterogeneity in primary gastric adenocarcinoma by single cell RNA sequencing. Gut.

[17] Tirosh I (2016). Dissecting the multicellular ecosystem of metastatic melanoma by single-cell RNA-seq. Science.

[18] Picelli S (2014). Full-length RNA-seq from single cells using smart-seq2. Nat. Protoc..

[19] Bolger AM, Lohse M, Usadel B (2014). Trimmomatic: A flexible trimmer for Illumina sequence data. Bioinformatics.

[20] Kim D, Langmead B, Salzberg SL (2015). HISAT: A fast spliced aligner with low memory requirements. Nat. Methods.

[21] Liao Y, Smyth GK, Shi W (2014). FeatureCounts: An efficient general purpose program for assigning sequence reads to genomic features. Bioinformatics.

[22] Zhou Y (2019). Metascape provides a biologist-oriented resource for the analysis of systems-level datasets. Nat. Commun..

[23] Zheng GX (2017). Massively parallel digital transcriptional profiling of single cells. Nat. Commun..

[24] Zhang X (2016). Single-cell analyses of transcriptional heterogeneity in squamous cell carcinoma of urinary bladder. Oncotarget.

[25] Tirosh I (2016). Single-cell RNA-seq supports a developmental hierarchy in human oligodendroglioma. Nature.

[26] Meacham CE, Morrison SJ (2013). Tumour heterogeneity and cancer cell plasticity. Nature.

[27] Chung W (2017). Single-cell RNA-seq enables comprehensive tumour and immune cell profiling in primary breast cancer. Nat. Commun..

[28] Patel AP (2014). Single-cell RNA-seq highlights intratumoral heterogeneity in primary glioblastoma. Science.

[29] Kim KT (2015). Single-cell mRNA sequencing identifies subclonal heterogeneity in anti-cancer drug responses of lung adenocarcinoma cells. Genome Biol..

[30] Du X (2014). Role of Notch signaling pathway in gastric cancer: a meta-analysis of the literature. World J. Gastroenterol..

[31] Kohno T (2012). KIF5B-RET fusions in lung adenocarcinoma. Nat. Med..

[32] Xu J, Gong L, Qian Z, Song G, Liu J (2018). ERBB4 promotes the proliferation of gastric cancer cells via the PI3K/Akt signaling pathway. Oncol. Rep..

[33] Quigley DA (2018). Genomic hallmarks and structural variation in metastatic prostate cancer. Cell.

[34] Takahashi N (2016). Prognostic role of ERBB2, MET and VEGFA expression in metastatic colorectal cancer patients treated with anti-EGFR antibodies. Br. J. Cancer.

[35] Li J (2017). Methylated claudin-11 associated with metastasis and poor survival of colorectal cancer. Oncotarget.

[36] Fidler IJ (2003). The pathogenesis of cancer metastasis: The 'seed and soil' hypothesis revisited. Nat. Rev. Cancer.

[37] Liu F (2016). Systems proteomics view of the endogenous human Claudin protein family. J. Proteome Res..

[38] Mansouri V, Rezaei Tavirani S, Zadeh-Esmaeel MM, Rostami-Nejad M, Rezaei-Tavirani M (2018). Comparative study of gastric cancer and chronic gastritis via network analysis. Gastroenterol. Hepatol. Bed. Bench.

[39] Zhao Z (2016). Evolution of multiple cell clones over a 29-year period of a CLL patient. Nat. Commun..

