# Supplementary material for: Comprehensive analysis of metastatic gastric cancer tumour cells using single-cell RNA-seq
Source: Sci Rep. 2021 Jan 13;11:1141. doi: 10.1038/s41598-020-80881-2 (PMC7806779; doi:10.1038/s41598-020-80881-2)
Supplement: Supplementary file 1 — Supplementary Figures. [file 41598_2020_80881_MOESM1_ESM.docx]

**Comprehensive analysis of** **metastatic gastric cancer tumour cells using single-cell RNA-seq**

Bin Wang^1^, Yingyi Zhang^1^, Tao Qing^2^, Kaichen Xing^3^, Jie Li^3^, Timing Zhen^3^, Sibo Zhu^2*^, Xianbao Zhan^1*^

1. Department of Oncology, Changhai Hospital, Second Military Medical University, 200433, China.
2. School of Life Sciences, Fudan University, Shanghai, 200438, China
3. Shanghai Cinoasia Institute, Shanghai, 200438, China

*Corresponding Author:

**Xianbao Zhan:** Department of Oncology, Changhai Hospital, Second Military Medical University, NO. 168 Changhai Road, Shanghai, China, 200433

Email: zhanxianbao@csco.org.cn

Tel: +86-21-31161441

**Sibo Zhu**: School of Life Sciences, Fudan University, NO.2005 Songhu Road, Shanghai, 200438, China.

E-mail: sibozhu@fudan.edu.cn

Tel: +86-21-61400037

**Supplementary figures**


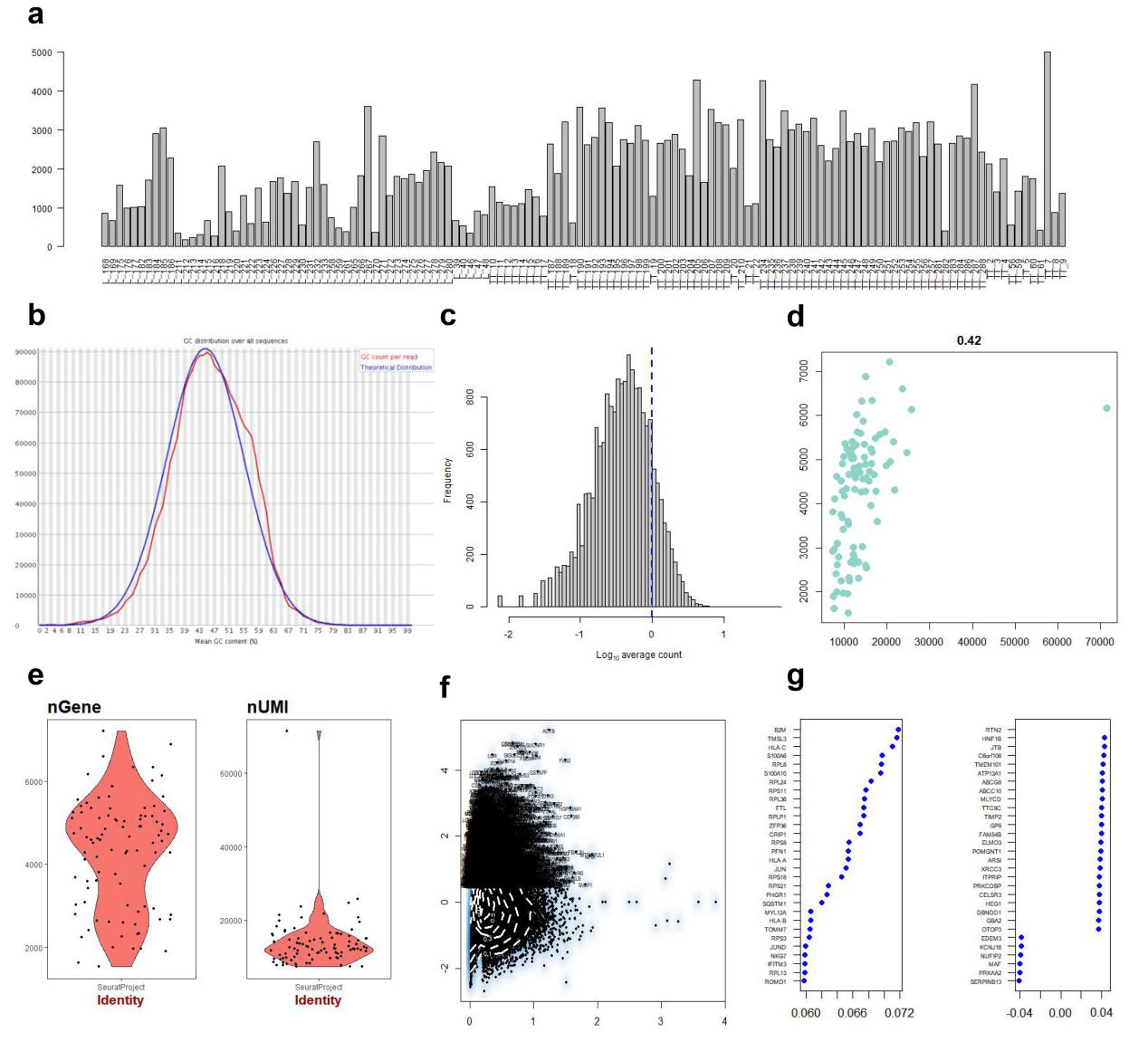


Figure S1. **Single cell data set quality control.** (a) Gene counts distribution was listed across all 617 single cells. (b) Histogram was plotted to show a highly concordance of GC content with theoretical distribution. (c) A normal count per cell distribution data was displayed. (d) Correlation between number of genes and counts. (e) Number of genes detected in all cells, TT samples and TP samples. (f) VariableGenes. (g) VizPCA


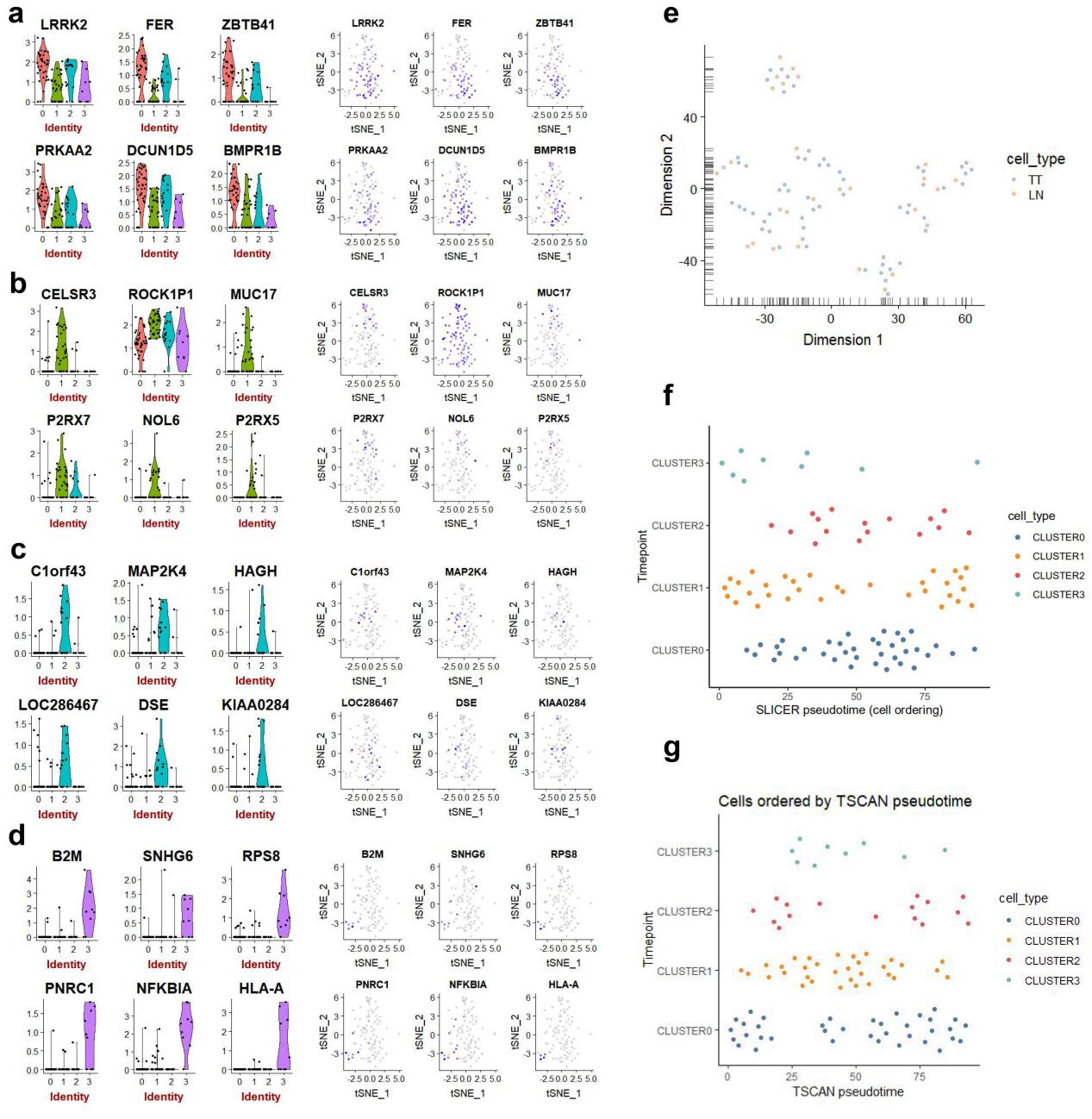


Figure S2. **Trajectory analysis of cell clusters.** (a-d) Top six markers in each group were labeled in red. (e) Stem cell markers were applied to identify. (f-h) SLICER, TSCAN and Diffusion map pseudotime tools revealed that four of the Gastric cancer cell.
